# Medical and Philosophical Intelligence

**Published:** 1811-12

**Authors:** 


### MEDICAL AND PHILOSOPHICAL INTELLIGENCE.

We have great pleasure in submitting to our readers, the Exordium to the Statutes of the Blenheim-Street Medical Society, which, instituted in the present year, already vies in numbers and respectability with other associations of longer standing; and from the zeal and enterprize of its members, promises to advance medical science.

Exordium.—An enlightened genius, the father of experimental philosophy, has declared that *knowledge is power;* and he has impressed succeeding generations with a conviction, that in natural science this knowledge results from observation, experiment, and the record of facts. If by pursuing the suggestions of the *Novum Organum*, an individual mind can, in any degree, penetrate into the secret laws of Nature, a union of minds employed on one subject, and impelled by the same views, may be expected still further to explore the operations of this mysterious principle. Upon this expectation have societies for the improvement of science been formed. And out of this, without arrogating extraordinary resources, or the ability to employ common means with unusual dexterity, has the Blenheim-Street Medical Society, also, arisen.

In

In the early part of 1811, some individuals who were ardent in the pursuit, if they were not deeply versant in the principles of science; and being convinced that by a communion of intellect, as by a combination of mechanic powers, effects may be produced beyond the reach of individual exertion, projected, and reduced to form, this society. Its progress, hitherto, might serve to evince, if it were required, that the light of knowledge may be elicited by collision of sentiment; and that the hope is neither remote nor doubtful, that from sources actually within its reach, or to arise out of probable contingencies, facts may be collected, which will assist to extend possibly, the *circle of Medical Science.*

The publication of its statutes may demand an exposition of the Society's principles, its means, and its discipline.

To meliorate the theory and the practice of the medical art generally; to afford the opportunity of improvement by the exercise of the mental faculty, individually, is the ground and basis of its views.

To unite Professors, Practitioners, and Students, under the definite classes of Fellows, and Honorary Associates, is the means the Society employs.

Its Fellows are those in whom the property of this Society is vested, from whom its funds emanate: who pass its decisions into laws, and who are subjected to censure, fine, and expulsion.

Its Honorary Associates are selected from amongst gentlemen whose standing, conduct, and acquirements in the profession, or in general science, entitle them to distinction. They are exempt from offices and fines: they have no interference with the business of the Society, except by request of the Fellows; but the Society may withdraw the distinction it has conferred by the vote of four-fifths of its Fellows.

On the principles, and by the statutes of the Society, its Fellows when removing to, or residing at, any place beyond a given distance from the station of the Society's meetings, in London, will have their privileges and duties modified. To its Fellows thus circumstanced, it is that the Society will look for valuable materials. The local knowledge of distant parts, the diseases of remote regions, natural history in all its branches, *materia medica*, both professional and popular, cannot fail to furnish subjects of high interest to the Society. Through this class the Society may expect, without incurring the charge of being visionary, to hold an intercourse with all parts of the world.

This opportunity to press on its Fellows, who may remove beyond the power or the convenience of personal attendance, what the Socity expects from their exertions, cannot be passed silently over. It is ardently wished that they will bear in mind that still they will continue to make a constituent portion of the Society; that they will participate in the credit it may attain; that at the station where the Society's meetings are held, they will be regarded with respect proportioned to their endeavours to serve its cause; and will

3 U 2 have have the honours awarded to them that their communications may demand, and the resources of the Society can admit.

The discipline of the Society will be founded, necessarily, on its code of statutes. These having been formed by the deliberation of its Fellows, revised and adjusted by its Council, will be found, it is hoped, at once perspicuous and compressed, without verbosity clear, and, though not neglectful of elegance, uniting plainness with precision.

While the Society looks to the great object, *improvement in Medical Science*, it has not been unmindful of the advantage of application, and of the benefit that will arise to its junior branches by cherishing a habit of arrangement and composition; and, by acquiring readiness and perspicuity from the practice of *viva voce* discussion. With this view a thesis or dissertation is required, *seriatim*, from each of its Fellows; and debate has been instituted under the direction and controul of a President, who, for the time being, is absolute.

Under this system, pursued with that steady perseverance, which the objects of the Society deserve, as far as human projects can, this promises to accomplish its views. But should performance never reach what expectation has held forth, disappointment will be moderated by reflecting that all the institutions of man are imperfect; yet as long as this society continues to be governed by candour, liberality, and a love for science, it will have the consciousness of endeavouring well; and its respectability will remain whilst its principles impel it to believe that the first and last determination, the alpha and omega of human intellect, should be directed to the discovery of truth.

---

*Case of Hydatids discharged by stool and vomiting.* (Hufeland's Journal).—A woman aged forty-one years, of rather an irritable constitution, but in general healthy, suffered for six years great distress, which occasioned her to fall into a feverish state. She drank during the heat of the fever a glass of cold water, from which, at first, she experienced a pain and pressure in the region of the stomach; she observed, at the same time, that it appeared to be swelled. The swelling daily increased; the appetite failed; and the patient complained of a sense of fulness after her meals, and a continual pain and oppression about the stomach. She especially felt uneasy after exercise. Several physicians had pronounced different opinions upon her complaint, and had employed various means without success. For upwards of a year the catamenia had become irregular, being often suspended for a long time, and then appearing in excess. This derangement in the menstrual flux did not, however, seem to be connected with the symptoms that have been detailed. In the month of June, last year, she was attacked, after a violent fright, with fever. This yielded to the remedies employed, but the patient's tongue continued covered with a white fur: she was affected with a disagreeable taste, and had frequent attacks of pain in the stomach. At the same time she was troubled with watchfulness, restlessness, lessness, and copious sweats. She took bitters and ether. The bowels were kept open by pills of assafœtida, aloes, soap, and castor, but without any evident benefit. She complained much at this period of a dull pain situated in the left side of the pit of the stomach: pressure upon it produced exquisite pain. She was affected with nausea, succeeded by vomiting, which, as well as the stools, gave vent to an abundance of viscous mucus. The disease had made her so irritable, that she was often enraged about the most trivial domestic occurrences. An emetic of ipecacuanha and tartarized antimony was prescribed; it occasioned vomiting, and the pain almost left her. The pills before mentioned seemed now to act with much advantage, but the amendment was not constant. Some days after, the patient complained again of severe pain in the region of the stomach, particularly towards the left side. She felt a sense of weight which she had not experienced before. It seemed to her as if, on certain motions of the body, a heavy mass pressed on the left and lower part of the epigastric region. She had no appetite, frequent nausea, and had become very feeble. Various remedies, both external and internal, were tried, till the occurrence of a phenomenon, very surprising to the physician, but very salutary to the patient. She felt an extraordinary desire to go to stool, and voided in one day, after several evacuations, sixteen bladders of various descriptions; some were burst, other entire; some were of the size of pigeons' eggs, others were nearly as large as hen's eggs. Most of them were of the oval form. The membrane which composed these bladders was thin, but so firm that those which were yet whole might be taken up with foreceps without danger of being broken. After being placed some time in water, the membrane might easily be separated into two lamina or leaves. Some of the bladders had a sort of pedicle, by means of which they probably attached themselves to the inner coat of the intestinal canal. In those which had not these pedicles, small round holes were observed, and these bladders appeared to have been detached from their pedicles. Some of them were filled with a limpid, transparent, gelatinous substance. On the following days, the patient again voided by stool about fifty more of these bladders. Several days after these evacuations, she experienced a disagreeable taste, with a stinking smell. Repeated vomitings occurred, in which, at first, she rejected bladders exactly resembling those voided by the anus, and a great quantity of viscous masses, which according to the patient's account were of the size and form of a boiled egg, and of a yellow colour: they diffused a fœtid stench. After these evacuations, she experienced a sense of emptiness in the region of the stomach, and in the left side of the abdomen, as if, according to her expression, the stomach and part of the entrails had been removed from her. Since this epoch she has been free from every inconvenience.

---

Died lately, at Gloucester, after a few days severe illness, Charles Brandon Trye, Esq. F. R. S. and senior surgeon to the Gloucester Infirmary. A man that will be long regretted by the thinking part of of that community; not only as a surgeon, but as a man extremely useful in various undertakings of national concern, such as rail-roads, canals, &c. in the planning of which he evinced great genius. As a surgeon, his practice was extensive, and his success great. Many arduous and difficult operations he performed, which ended in perfect cures, after others of eminence had shrunk from the task. His operations were conceived and executed from a perfect knowledge of the structure of the human body, attained by a well grounded education, and constant intense study through life. He was educated under the eminent surgeon, Mr. Russell, of Worcester, then with John Hunter, was house-surgeon to the Westminster Infirmary, and afterwards assistant to the very ingenious and scientific Sheldon. He was for some time house-surgeon and apothecary to the Infirmary in Gloucester. Shortly after he quitted that situation, he was elected surgeon to that charity, an office which he filled for near thirty years, discharging its duties with great credit to himself; while those placed under his care were sensible of the advantages they possessed from his assiduous attention to their sufferings. He trained up several surgeons, many of whom are exercising the medical profession in various parts of the kingdom, with credit to their preceptor, honour to themselves, and utility to mankind. As an author he was well known in the literary part of the medical world. He published a reply to Jesse Foot's attack upon the Practice and Writings of John Hunter.—Observations on Retention of Urine.—An Essay on the Swelling of the Lower Extremities, incident to Lying-in Women.—Illustrations of some of the Injuries to which the Lower Limbs are exposed.—Essay on some of the Stages of the Operation of Cutting for the Stone.—And several papers of a miscellaneous nature connected with the profession, in various periodical publications. He was a steady friend and promoter of the vaccine inoculation.

---

Dr. Metternich has lately used the *rhododendrum chrysanthum* in gout. Koplin had already given a decoction of the stem and leaves. In this form it produced vomiting, hypercatharsis, and vertigo. Dr. Metternich gave the powder in doses of ten to forty grains in the day, at three or four times, according to the strength and sensibility of the patients. The remedy should be continued several weeks. In some cases the gout was radically cured by it. It also proved useful in rheumatic pains, which began with fever and swelling of the hands and feet.

---

The operation for the varicous *Vena Saphena major* has very lately received considerable improvement. The mode of operating formerly employed was too frequently followed by fatal results. The present method, the improvement of which consists in removing the ligature from the vein in a few minutes after its application, we are assured is always effectual to obliterate the canal of the vessel, and has not yet been succeeded by any untoward circumstance. We are informed that Mr. Freer of Birmingham has employed the operation, thus modified, several times with complete success.

*Continued from page* 420.—Tables of the deaths by Small-pox in London, in the twelve years preceding the practice of Vaccination, and in the twelve years immediately succeeding the introduction of that disease.

1. Deaths by Small Pox preceding Vaccination in the first four years.

| | | | |
|---|---|---|---|
| 1 | In | 1787 | 2418 |
| 2 | | 1788 | 1101 |
| 3 | | 1789 | 2077 |
| 4 | | 1790 | 1617 |
| | | | 7213 |

*In the second Four Years.*

| | | | |
|---|---|---|---|
| 1 | In | 1791 | 1747 |
| 2 | | 1792 | 1563 |
| 3 | | 1793 | 2382 |
| 4 | | 1794 | 1913 |
| | | | 7610 |

*In the third Four Years.*

| | | | |
|---|---|---|---|
| 1 | In | 1795 | 1040 |
| 2 | | 1796 | 3548 |
| 3 | | 1797 | 522 |
| 4 | | 1798 | 2237 |
| | | | 7347 |

2. Deaths by Small Pox during the Vaccine practice, in the first four years.

| | | | |
|---|---|---|---|
| 1 | In | 1799 | 1111 |
| 2 | | 1800 | 2409 |
| 3 | | 1801 | 1461 |
| 4 | | 1802 | 1579 |
| | | | 6560 |

*In the second Four Years.*

| | | | |
|---|---|---|---|
| 1 | In | 1803 | 1202 |
| 2 | | 1804 | 622 |
| 3 | | 1805 | 1685 |
| 4 | | 1806 | 1158 |
| | | | 4667 |

*In the third Four Years.*

| | | | |
|---|---|---|---|
| 1 | In | 1807 | 1297 |
| 2 | | 1808 | 2257 |
| 3 | | 1809 | 1163 |
| 4 | | 1810 | 1198 |
| | | | 5915 |

| | |
|---|---|
| The total number of deaths by Small Pox in twelve years previously to Vaccination, amounts to - - | 22,170 |
| Ditto, subsequently to Vaccination - - | 17,122 |
| | 5,048 |

the number of deaths in the first twelve exceeding the number in the twelve succeeding during vaccination, *i. e.* about 420 persons per annum fewer for twelve years died since, than before Vaccination.

---

Dr. Wigan of Hamburgh has employed the following remedy, with great success, in croup. According to the age of the patient, he gives two, three, four, or five grains of calomel, with half a grain, or a whole grain of musk, every hour till vomiting of mucous matter takes place. This generally occurs after the third dose. The matter vomited has the appearance of greenish-yellow cream, of puriform consistence. As soon as the vomiting is excited, there is reason to expect a successful issue, and the powder should then be given only every two or three hours. At the same time, with the intention of favouring the vomiting, Dr. Wigan gave every hour, or every two or three hours, two or three table spoonfuls of a mixture ture of oxymel of squills, syrup of senega, sal ammoniac, and antimonial wine of Huxham. Mercurial ointment was also applied to the neck and upper part of the chest and shoulders; and a warm bath every six or eight hours.

---

On Wednesday, October 2d, 1811, the Society for Relief of Widows and Orphans of Medical Men in London and its Vicinity, held their half-yearly general court at the usual place of meeting, the Gray's-inn coffee-house, Holborn; when the annual election of Officers, and Directors took place, and the following gentlemen were elected into office for the year ensuing.

Patron, His Royal Highness the Duke of Kent.

President, James Ware, Esq. F. R. S. &c.

Vice-Presidents, Sir F. Milman, Dr. Garthshore, Dr. Lettsom, Dr. Blane, Dr. Squire, Dr. Dennison, Sir W. Blizard, Mr. Heaviside, Mr. Howard, Mr. Nevinson, Mr. Moore, and Mr. Rendall.

Treasurers,—Dr. Denman, Dr. John Sims, Dr. Dennison.

Directors.—*Physicians*, Dr. Frampton, Dr. Walshman, Dr. S. H. Jackson, Dr. Marcet, Dr. R. Pearson, Dr. Croft, Dr. Haworth, Dr. Adams.—*Surgeons*, Mr. Milward, Mr. Steele, Mr. H. L. Thomas, Mr. C. M. Clarke, Mr. Ramsden, Mr. Lewis, Mr. Tho. Blizard, Mr. Chevalier.—*Apothecaries*, Mr. Coates, Mr. Seaton, Mr. Starr, Mr. Malim, Mr. Pilliner, Mr. Moore, jun. Mr. Wheeler, and Mr. Hunter.

Secretary,—Mr. Wm. Chamberlaine.—Collector,—Mr. Geo. Hunt.

This Society was instituted in the year 1788. Its capital is now eighteen thousand four hundred pounds, three per cent. consolidated Bank annuities, and two hundred pounds Navy five per cents. out of the interest of which, down to the 18th of September in the present year, 1811, the sum of three thousand three hundred and forty-eight pounds, three shillings, has been distributed among the Widows and Orphans of deceased Medical Men, Members of this Society, many of whose families have been left without any provision whatever.

---

A very ingenious and important improvement has lately been made on the Midwifery forceps, by Mr. James Davidson, Surgeon, in Perth, which consists in locking the two ends thereof round the neck of the child in the uterus, hitherto deemed impracticable; by means of which, the instrument is prevented from the possibility of slipping, the head entirely surrounded and secured, and the child extracted with ease, certainty, and expedition, in all head cases of difficult labour. It has been already tried in several instances with complete success, and may be the means of saving many lives.

---

Hufeland has lately recommended belladonna in hooping-cough. It has often produced favourable effects in a few days, in cases even where opium and musk had been inefficacious. The dose for children from three to six years, is a quarter of a grain night and morning; in some cases the dose may be increased and repeated oftener.

---

*Russel Institution.* Mr. George Singer will commence a course of Lectures on Electricity at this Establishment, towards the close of December. These Lectures will include the History, and Practice of the Science; its application to the Solution of natural Phenomena and the promotion of Chemical knowledge. They may be considered as a popular abstract of an extensive series delivered last year at the Scientific Institution, of which some account is given in a former volume of this Journal.

---

*Dr. Squire's Lectures.*—Dr. Squire will on Saturday, December 14, begin a Course of Lectures on the Principles and Practice of Midwifery, and the Diseases of Women and Children.

